# L1 in tumor vasculature

**DOI:** 10.18632/oncotarget.7791

**Published:** 2016-02-29

**Authors:** Ugo Cavallaro

**Affiliations:** Department of Experimental Oncology, European Institute of Oncology, Milano, Italy

**Keywords:** L1 cell adhesion molecule, tumor angiogenesis, vascular normalization, gene expression

Angiogenesis is a pre-requisite for the growth and the metastatic dissemination of solid tumors. Therefore, interfering with the angiogenic process has long been proposed as a therapeutic strategy to restrain cancer progression [1]. In this context, drugs that inhibit vascular endothelial growth factor (VEGF) signaling have proven efficacious in different tumor types, especially when combined with conventional chemotherapeutics or targeted therapies, and have therefore entered the clinical practice. It should be noted, however, that in most cases VEGF-targeted therapies prolong patients’ disease-free and/or overall survival by just a few months. Moreover, both experimental data and clinical observations suggested that tumors activate escape or evasion mechanisms in response to VEGF inhibition, acquiring more aggressive properties [1]. Taken together, these findings emphasize the urgent need to investigate in more detail the biological mechanisms and the molecular players involved in tumor angiogenesis, aimed at defining novel vascular-targeting strategies that may help overcome these problems.

We have recently reported a series of observations that point to the protein L1 as a candidate target for such strategies. L1 (also known as L1CAM or CD171) is a cell surface molecule that belongs to the immunoglobulin superfamily of cell adhesion molecules, and has been extensively characterized as an important player in the development and plasticity of the nervous system [2]. Furthermore, several studies have shown the aberrant expression of L1 in cancer cells, where it promotes invasion and metastasis and its level correlates with poor prognosis [3]. Screening a panel of different tumor types, we observed that L1 is expressed in cancer-associated vasculature, while no or very little expression was detected in normal vessels [4, 5]. To address the question of whether L1 plays any role in tumor vessels, we combined the genetic inactivation of endothelial L1 in Tie2-Cre;L1floxed mice with a syngeneic, orthotopic model of pancreatic carcinoma. Tumor growth and dissemination were markedly reduced in Tie2-Cre;L1floxed mice as compared to their control littermates. The decrease in tumor burden was accompanied by a significant reduction in microvessel density, causally implicating endothelial L1 in tumor angiogenesis. Indeed, our *in vitro* studies on genetically manipulated endothelial cells revealed that L1 is necessary and sufficient for cell proliferation, migration and tube formation. Unexpectedly, L1 also exerted a regulatory function on tumor vessel architecture and function. L1-expressing tumor vessels showed the typical alterations of cancer vasculature, with poor pericyte coverage and basement membrane deposition, loss of endothelial polarity and enhanced permeability. In contrast, L1-deficient vasculature exhibited an almost “normal” phenotype, as shown by pericyte coverage, production of a continuous basement membrane and endothelial cell polarity. This resulted in the abrogation of tumor vessel permeability, indicating that L1- dependent regulation of vessel architecture has functional implications [5].

This set of results, together with a series of observations on genetically manipulated endothelial cells in culture [5], pointed to L1 as a master regulator of the tumor vasculature, and indicated that L1 plays a pivotal role not only in cancer-associated neovascularization, but also in the aberrant morphological and functional features of tumor vessels. Mechanistically, such a pleiotropic function implied the ability of L1 to control key molecular pathways within the endothelium. Indeed, our data showed not only that L1 exerts a massive regulation of the endothelial transcriptome, but also that such a regulation involves factors that play a prominent role in angiogenesis, such as VEGF-A, VEGF-C and Dll4, as well as molecules that contribute to endothelial-mesenchymal transition, such as Zeb-1, Zeb-2, N-cadherin, S100A4, etc.. In addition, the IL6/JAK/STAT3 pathway was found to be an important effector downstream of L1 [5].

Besides shedding light on novel mechanisms causally linked to the dysregulated architecture and function of cancer vessels, our data suggested that interfering with the function of vascular L1 might represent an innovative therapeutic option. Indeed, we observed that treating tumor-bearing mice with L1-neutralizing antibodies recapitulated the genetic inactivation of endothelial L1, with decreased tumor growth and angiogenesis accompanied by vascular normalization [5]. Future studies should aim at comparing L1-targeted therapies with classical antiangiogenic treatments and at exploring possible synergistic effects. It would be of particular relevance to test whether neutralizing vascular L1 allows overcoming the evasion and escape mechanisms that are observed in tumors treated with anti-VEGF therapy (see above). Our data also imply that targeting L1 might prove particularly efficacious in those tumors in which L1 is found not only in the vessels but also in malignant cells [3], due to the possibility of interfering simultaneously with L1-driven tumor neovascularization and invasion.

Antiangiogenic drugs are commonly used in combination with conventional chemotherapeutics or targeted therapies and it can be anticipated that combined strategies will also represent the best option for L1- based treatments. Besides the obvious expectation of an additive effect between the cytotoxicity towards neoplastic cells and prevention of tumor neovascularization, it is tempting to speculate that the vascular normalization promoted by L1 inactivation, by restoring a more uniform blood perfusion of the tumor tissue, might promote a better distribution of the anti-neoplastic drugs, thus enhancing the therapeutic response. Indeed, despite this remains a controversial issue, the hypothesis that vascular normalization improves the clinical efficacy of chemotherapy is supported by a growing body of evidence [6].

Thus, L1 is emerging both as a key player in the orchestration of vascular pathophysiology associated to cancer development and as a promising target for innovative therapeutic strategies targeting tumor vessels. Future preclinical studies will give further insights into the feasibility and the optimal applications of L1-based antitumor treatments.
